# The impact of food marketing via video game live streaming on snack intake in adolescents: a randomised controlled trial

**DOI:** 10.1017/S1368980025100487

**Published:** 2025-06-03

**Authors:** Rebecca Evans, Paul Christiansen, Andrew Jones, James Finney, Emma Boyland

**Affiliations:** 1 Department of Psychology, University of Liverpool, Eleanor Rathbone Building, Bedford Street South, Liverpool L69 7ZA, UK; 2 School of Psychology, Liverpool John Moores University, Tom Reilly Building, Byrom Street, Liverpool L3 3AF, UK

**Keywords:** Randomised controlled trial, Food marketing, Video game live streaming, Adolescents, Snack intake

## Abstract

**Objective:**

The marketing of foods and non-alcoholic beverages (hereafter: food) high in fat, salt and/or sugar (HFSS) is implicated in the development of poor dietary habits, overweight and obesity. Digital media, including video game live streaming platforms (VGLSP), are an increasingly prominent source of food marketing exposure, particularly for young people. This study aimed to experimentally examine the impact of food marketing via VGLSP on eating behaviour in young people.

**Design:**

A between-subjects randomised controlled trial design was used to explore the impact of exposure to HFSS food marketing in a video game live stream (a static food banner advert present throughout the footage) on immediate consumption of the marketed snack and an ‘alternative brand’ of the same snack in a sample of adolescents (*n* 91, *M*_age_ = 17·8, 69 % female). Relationships with food-advertising-related attentional bias and inhibitory control in relation to branded food cues were also examined.

**Setting:**

University Psychology laboratory.

**Results:**

Exposure to HFSS food marketing, compared with non-food marketing, did not significantly impact immediate marketing or overall snack intake. Additionally, no significant effects for attentional bias or inhibitory control were found. However, although the overall model was non-significant, greater weekly use of VGLSP was significantly associated with greater marketed snack intake.

**Conclusions:**

Findings suggest that while acute exposure to food marketing in VGLSP did not impact snack intake, perhaps more sustained exposure is impactful. Further exploration of this effect is needed, as well as studies investigating the potential impacts of other food marketing formats within VGLSP.

The marketing of high in fat, salt and/or sugar (HFSS) food has been implicated in the development of poor dietary habits, overweight and obesity, which has serious consequences for health^(1)^. In recent years, major food brands have expanded their focus from traditional media to digital media as an outlet for food marketing^(2)^. This focus has included new and emerging digital platforms, such as video game live streaming platforms (VGLSP)^(3,4)^.

An influencer is an individual with a large following in one or more niches (e.g. gaming). A VGLSP is a platform where streamers or ‘gaming influencers’ can broadcast live video game content to their audience online. The top VGLSP globally is Twitch, which has 78 % of the market share in terms of hours watched^(5)^, and averages over 2·8 million concurrent viewers^(6)^. A large majority of UK adolescents use video-sharing platforms (98 %), play video games online (76 %) and watch live streams (73 %)^(7)^. Streamed games on Twitch also include those popular with young people, such as Fortnite and Minecraft^(5,8)^.

Streamers advertise brands and products, often simultaneously, in a variety of ways on VGLSP. Common in-stream advertising placements include jersey patches (i.e. brand logos on clothing), product placements (e.g. a streamer consuming a product) and banners (i.e. digitally overlaid images that can be static or have moving effects applied)^(9)^. In-stream banners are always visible and unobstructed by gameplay^(9)^. The most frequently marketed food categories (and brands) on Twitch are energy drinks, fast food restaurants, fizzy drinks and processed snacks^(3,4)^.

Recent meta-analyses have demonstrated that exposure to unhealthy food marketing via television and digital media (social media, advergames) is associated with significant increases in children and adolescents’ marketed and overall ad-libitum HFSS food intake, relative to no or non-food marketing^(10–12)^. However, the impact of food marketing via VGLSP specifically is less clear. There is cross-sectional evidence that recall of food marketing on VGLSP is associated with greater consumption of marketed HFSS foods in both 13–18-year-olds and a predominantly young adult (18–24-year-old) sample^(13,14)^. Further, a meta-analysis exploring digital marketing techniques of particular relevance to VGLSP (digital game-based and social media influencer marketing) found that exposure was associated with significant increases in children and adolescents’ marketed and overall HFSS snack consumption, relative to no or non-food marketing^(15)^. However, to date, there is no experimental research exploring the impact of food marketing via VGLSP on immediate snack consumption in young people. It is crucial that we understand how food marketing in various innovative digital spaces impacts food intake to inform evidence-based policies on unhealthy food marketing.

Adolescents may be particularly vulnerable to the effects of food marketing^(16)^. Unique age-related developmental vulnerabilities include peer-group influence and identity formation processes^(16)^. Neurobiologically, psychological mechanisms involved in the regulation of eating behaviour, such as neural substrates of inhibitory control, are not fully developed until late adolescence, and reward sensitivity is heightened at this age^(16)^. This means that adolescents may lack the cognitive resources and motivation to inhibit appetitive responses. Marketing of HFSS foods to adolescents is specifically designed to take advantage of these unique developmental vulnerabilities^(16)^. For example, adolescents are targeted by digital marketing that is disguised as entertainment (e.g. via social media influencers and digital games) which is more difficult to recognise as advertising and resist^(16)^. Despite this susceptibility, much of the literature on youth food marketing focuses on children (only 18 % of existing studies focus exclusively on teenagers)^(17)^.

There is also a need to better understand the psychological mechanisms underpinning food marketing effects, particularly regarding contemporary digital food marketing and young people. The dual-process model of cognition is a useful theoretical framework for the examination of these psychological mechanisms^(18)^. The model is made up of two separate yet related cognitive processes: attentional bias and inhibitory control. Attentional bias is defined as the extent to which a cue (e.g. a food advert) grabs and holds attention. Inhibitory control refers to the ability to suppress reward-driven behaviour (e.g. consuming appetitive food). The addiction literature demonstrates that alcohol and tobacco advertising prime automatic consumption, and that young people are less able to inhibit this drive to consume^(19,20)^. However, research exploring this effect in relation to food marketing is limited.

Existing evidence supports the notion that food marketing would impact an individual’s cognitive processes. A meta-analysis found considerable evidence that attentional bias towards food-related stimuli is associated with food craving and intake^(21)^. Moreover, children with a higher gaze duration (i.e. greater attention bias) for food cues in an advergame ate more advertised snacks^(22)^. In relation to Twitch users (aged 18–24), higher scores on external food cue reactivity measures were related to increased odds of craving products seen on Twitch^(23)^. Overall, this suggests that heightened sensitivity to food-related cues (e.g. food marketing) could play a causal role in overeating.

Regarding inhibitory control, impairments in the ability to suppress reward-driven behaviour are associated with the choice and intake of HFSS foods and obesity^(24,25)^. Additionally, children who participated in a go-no-go food task in which the advertised food was consistently associated with no-go cues (strengthening inhibitory control) were found to consume significantly fewer calories after playing an advergame promoting energy-dense foods, compared with those who did not participate in the task^(26)^. Therefore, it would be logical to assert that inhibitory control interacts with cues from obesogenic environments (e.g. food marketing).

Together, this evidence suggests that increased attentional bias towards food-related cues and poor inhibitory control are likely to be associated with increased cue-induced food consumption, as predicted by the dual process model of cognition. The collective effect of attentional bias and inhibitory control on food marketing-induced eating is yet to be tested. Moreover, existing studies assessing their individual impacts have been restricted to food marketing via advergames. Given that recent neuroimaging evidence shows that different advertising mediums have unique effects on neural responses to food cues in children^(27)^, medium-specific food marketing research is warranted.

The current study used a between-subjects randomised controlled trial to primarily explore the effects of HFSS food marketing exposure via a mock Twitch stream on immediate snack consumption in a sample of UK adolescents. The potential moderating role of attentional bias and inhibitory control was also assessed using computer-based behavioural tasks. The impact of several potential covariates (e.g. gender, habitual use of VGLSP) on snack consumption was assessed using questionnaire measures.

Our primary hypothesis was that participants in the experimental condition would have greater marketed snack intake (kcal) and greater overall snack intake (kcal) than those in the control condition. We also tested exploratory hypotheses: (i) participants with longer gaze duration towards the food advert would have a greater marketed snack intake and overall snack intake, (ii) those with poorer inhibitory control scores would have a greater marketed snack intake and greater overall snack intake in response to the food advert and (iii) the interaction between attentional bias and inhibitory control would predict marketed and overall snack intake (specifically, that individuals with greater attentional bias and poorer inhibitory control would consume the most food in response to the food advert).

## Methods

The study protocol was pre-registered, including design and analytic plans. The protocol, experiment materials and data can be accessed at https://osf.io/u6my3/.

### Participants

Sample size was calculated using G * Power. Based on a medium-large effect size of *d* = 0·6 (related studies exploring the impact of exposure to HFSS food marketing via social media influencers^(28)^ and advergames^(22)^ identified a medium-large sized effect on subsequent HFSS food intake) at 80 % power, the total required sample size for a between-subjects linear model with two groups and two covariates = 90. While no previous studies have examined the impact of experimental exposure to food marketing via videogame live streaming and potential moderators, it is likely that exploratory two- and three-way interaction effects (e.g. condition*gaze duration*inhibitory control on intake will be smaller, and therefore, the study may be underpowered to detect them)^(29)^. Participants were identified through direct contact with youth-focused organisations, word-of-mouth and social media. The inclusion criteria for participants were 13–18 years of age, no medical condition(s) which would have meant they could not abstain from eating for 2 h (e.g. diabetes), no food allergies or intolerances, not dieting to lose or maintain weight and no current or historical eating disorders. Data were collected January–August 2023 until the required sample size was reached.

### Design

The study was a between-subjects randomised controlled trial. Participants were allocated a participant number and, using a block randomisation schedule (in blocks of 10; www.randomizer.org), were assigned to either the control (non-food marketing) or experimental (food marketing) condition. Therefore, the independent variable was the marketing condition. Dependent variables were marketed (Doritos) and overall snack consumption (kcal). Covariates included scores on attentional bias and inhibitory control measures.

### Materials

#### Mock Twitch streams

One Twitch streamer, 31-year-old male Tyler ‘Ninja’ Blevins (https://www.twitch.tv/ninja), was selected based on his popularity with young people^(8)^. Therefore, this content was deemed likely to be typical of what adolescents may encounter when using Twitch or other similar VGLSP.

One video (high definition, 1280 × 720 pixels) of Ninja playing Fortnite from 27 July 2022 was obtained from Ninja’s Twitch channel using the screen-recording and video editing software Camtasia (TechSmith, MI, US). The video was cropped so that it was 7 min in duration (5 min of exposure is sufficient to prompt an intake effect in children^(12)^). Editing was also used to overlay a static banner advert designed for the study on each video for the full duration. Both versions of the video were identical but for the brand and product featured in the banner advert. In the control videos, the banner advert featured a non-food item (Adidas trainers), and in the test videos, it was an HFSS snack (Doritos Lightly Salted crisps). Processed snacks are the most frequently marketed non-perishable food category on Twitch with Doritos representing the top brand in this category^(3)^. Doritos is also the second leading snack brand in the UK and was therefore likely to be a familiar brand for our sample^(30)^. Similarly, Adidas is well-known by the UK public^(31)^. Lightly salted crisps were selected as they are suitable for vegetarians and vegans. No other marketing is featured in the videos. Each banner advert featured the caption ‘Ninja x [brand]’, which is often used to designate an influencer × brand collaboration. Visually, this advert is similar to in-stream banners used on Twitch (e.g. size, location)^(9,15)^. See the Appendix for the video and advertising stimuli used.

#### Cover story

Eating is a psychological process that can be modified by individuals if they are aware of the aims of the study^(32)^. Therefore, participants were informed that the study aimed to explore participant memory in relation to video game live streams and determine which streamers are the most engaging to watch. Some dummy questions were included in the questionnaire (e.g. ‘What colour hoodie was Ninja wearing?’) to make the cover story seem more authentic. The true aims of the research were not made explicit to the participants until after they completed the study.

#### Questionnaire

To adjust for potential effects on kcal food intake, a pre- and post-exposure questionnaire was created. Questionnaire measures were developed and delivered using the web-based survey tool Qualtrics XM.

### Pre-exposure

#### Demographics

Participants were asked their age, gender, ethnicity and residential postcode as a proxy for socio-economic advantage. Postcode was used to calculate their index of multiple deprivation decile (https://www.fscbiodiversity.uk/imd/). A decile of 1 means the postcode is in the bottom 10 % of the deprivation index.

#### Time spent on video game live streaming platforms per week

Participants were first asked ‘Have you ever used a VGLSP? For example: Twitch, YouTube Gaming, Facebook Gaming’. If [yes], participants were asked how many hours they spend on VGLSP (a) on a typical weekday (e.g. a Monday) and (b) on a typical weekend day (e.g. a Saturday). Total weekly hours were calculated as (typical weekday hours × 5) + (typical weekend day hours × 2).

#### Test brand liking

Liking of the test food was measured using 100-mm visual analogue rating scales anchored with 0 = really dislike and 100 = really like. Questions followed the format of ‘how much do you like [brand]?’. The test brands (Doritos, Tesco, Adidas) were included alongside other brands that did not feature elsewhere in the study (Nike, Cadbury’s) to disguise study aims. The other brands were selected based on being well-known by the UK public.

#### Hunger

Participants were asked ‘How hungry do you feel right now?’ which was responded to on a visual analogue rating scale anchored with 0 = not at all hungry and 100 = extremely hungry.

#### Prior streamer and video game familiarity

Participants were asked ‘How familiar are you with… (i) the Twitch streamer ‘Ninja’? and (ii) the videogame Fortnite?’ which was responded to on a separate visual analogue rating scale anchored with 0 = very unfamiliar and 100 = very familiar.

### Post-exposure

#### Liking of the stream

Participants were asked ‘How much do you like the Twitch stream that you just watched?’ which was responded to on a visual analogue rating scale anchored with 0 = really dislike and 100 = really like.

#### Awareness of marketing

Participants were asked ‘Did the Twitch stream you watched today have an advert in it?’ with a yes/no response. If [yes], participants were prompted to indicate the brand/product that was advertised. This question was embedded amongst several ‘dummy’ questions, consistent with the cover story. Marketing awareness was operationalised as whether the brand and/or product was correctly identified (0 = no, 1 = yes).

#### Awareness of study aims

Participants were asked ‘What do you think the aim of the study was?’ with a free-text response. A response was deemed correct if it referenced the impact of food marketing on snack consumption (or something to that effect). Awareness of study aims was operationalised as whether the true aim was correctly identified (0 = no, 1 = yes).

#### Behavioural tasks

Computer-based behavioural tasks were conducted on a desktop computer using Gazepoint v.5.1.0 and Inquisit v.5.0.14.0.

#### Attentional bias

A portable research-grade eye tracker (GP3 Eye Tracker, Gazepoint) was used to test attentional bias towards the banner advert in the test video. Data were obtained using a machine-vision camera with a 60 Hz sampling rate and 0·5–1 degree of visual angle accuracy. The two versions of the test video were uploaded to Gazepoint, and an area of interest was drawn around the banner advert. The test video was presented on a screen with a 1920 × 1080 resolution (16:9 ratio). Participants were seated 55 cm from the display screen and an adjustable chinrest was used to reduce head movement. A nine-point calibration system was used to calibrate the eye tracker prior to testing, in which an expanding–contracting circle appeared in every position on a screen-wide 3 × 3 grid of calibration points. Participants were asked to fixate on the circle. The calibration was repeated if any points did not calibrate successfully. Attentional bias was measured as the duration of fixations on the area of interest (i.e. gaze duration).

#### Inhibitory control

A cue-specific version of a stop-signal task originally developed by Logan *et al*.^(33)^ was used to measure inhibitory control. The task was programmed using Inquisit (https://mili2nd.co/l5ac). Participants were required to press the ‘D’ key in response to food images, and the ‘K’ key in response to neutral images. However, in some trials, a red ‘X’ (i.e. a stop signal) appeared over the image shortly after it appeared, which indicated that the response should be withheld. Sixteen images of branded foods and sixteen images of neutral objects (musical instruments) (see Appendix for images used) were included. Images of branded foods were selected based on the most frequently advertised food brands on Twitch^(3)^. This was because we were interested in inhibitory control in relation to HFSS foods that are commonly marketed on Twitch (i.e. ‘gamer’ foods) specifically. The test brand (Doritos) was not included, to minimise specific priming effects on eating^(34,35)^. Images of musical instruments were matched to branded food images in terms of size and colour. The task had 400 trials (200 food images, 200 neutral images), and a stop signal was presented in 25 % of the trials, consistent with recommendations to ensure reliability^(35)^. Stop signal reaction time (SSRT) was calculated using the integration method, and the difference in task performance (SSRT) between food and neutral pictures was calculated as SSRT food cues – SSRT neutral cues, with positive values indicating worse inhibitory control towards food cues. Participant scores were excluded if specific parameters were violated^(36)^.

#### Energy intake

Consistent with previous studies^(28)^, to measure energy intake, participants were told they could have a 5-minute ‘snack break’ and invited to eat *ad libitum* from two bowls of tortilla crisps). Each bowl contained 100 g of Doritos Lightly Salted tortilla crisps, but one was labelled ‘Doritos’ and the other was falsely labelled ‘Tesco’s’ (the largest supermarket chain in the UK). Participants were verbally informed of the purported brand difference. This approach, used in a similar study^(28)^, enabled brand-specific intake effects to be disentangled from any general consumption effects of the marketing. The quantity of crisps (100 g in each bowl) was used to avoid ceiling effects (i.e. participants consuming all the available crisps) and has been used for HFSS food provided to children in previous food intake studies^(28)^. Crisps were presented in plastic serving bowls and discreetly weighed pre- and post-intake (out of sight of the participant) to the nearest 0·1 g using a calibrated food weighing scale (Model CPA4202S; Sartorius AG, Germany). Intake was measured by calculating changes in snack vessel weight. Data were then converted into kilocalories (kcal) based on the manufacturer’s nutritional information.

#### BMI

Participant weight was measured to the nearest 0·1 kg with a calibrated weighing scale (Seca, model: 888, Germany), and height was measured to the nearest 0·1 cm using a stadiometer (Leicester Height Measure). BMI was calculated as kg/m^2^ and converted to a standardised Z score using WHO reference data^(37)^. BMI z-score outliers (< –4 sd or > 8 sd) were excluded based on the definition from Freedman *et al*.^(38)^.

#### Procedure

The study took place in a Psychology laboratory at the University of Liverpool. All participants attended one 45-minute session between 09.00 and 17.00. Informed consent was gathered prior to the session via Qualtrics. Firstly, participants completed a medical history questionnaire to confirm that they did not have any food allergies or intolerances. Then, the pre-exposure questionnaire was administered. Secondly, inhibitory control was assessed using the stop-signal task. The researcher read the instructions to the participants and gave them the opportunity to ask questions if they were unsure. After the task was complete, participants were asked to use the chin rest, and the height was adjusted accordingly. The eye-tracker was then set up, and participants were told that they would be watching a short excerpt from a Twitch stream, and that they should try to pay attention as they would be asked questions about it later. The researcher then played the appropriate version of the Twitch stream.

Post-exposure, participants were told that they could have a snack break. They were presented with the bowls of crisps and informed that they could eat as much or as little as they wanted. The researcher played a neutral Twitch stream (no marketing exposure) on the desktop computer and informed participants that they would not be asked questions in relation to this stream. The researcher then left the room for 5 min. After this period, the researcher returned and collected the bowls of crisps, which were reweighed in a separate kitchen area.

Participants then completed the post-exposure questionnaire. Following this, participant height and weight (without shoes) were measured and recorded. After the session was complete, participants were debriefed and informed of the true aims of the study. Participants (and any attending parent) were given a £5 Amazon voucher as compensation for their time. Participants were also given the option to enter a prize draw to win a £100 Amazon voucher. The winner was selected using a random number generator and their voucher was emailed to them.

### Data analysis plans

All analyses were conducted using R. Statistical tests’ level of significance was set at *P* < 0·05 for main analyses and *P* < 0·01 for sensitivity analyses.

We used two linear models to assess the impact of the condition (food marketing *v*. non-food marketing) on Doritos intake (model 1) and overall intake (model 2). We also assessed the impact of condition*gaze duration, condition*SSRT and condition*gaze duration*SSRT interactions in both models. To assess the assumptions of linearity and homoscedasticity and identify influential cases, Residuals *v* Fitted, Q–Q Residuals, Scale-Location and Residuals *v.* Leverage (Cook’s distance) plots were consulted. To assess the normality of residuals, residual histograms and Kolmogorov–Smirnov tests (*P* > 0·05) were inspected. Finally, multicollinearity between predictors was examined using VIF (Variance Inflation Factor; VIF > 5 is slightly problematic, VIF > 10 is very problematic^(39)^). For both models, sensitivity analyses were performed by replicating the main analyses (i) after excluding participants aged 19 years and over and (ii) after excluding aim guessers.

## Results

### Final sample

Ninety-five participants took part in the study (see Table 1 for sample characteristics). Eighteen participants indicated that they were 13–18 years old when answering the age screening question (pre-session) but reported a higher age when asked for their specific age in years and months during the lab session. Four participants were excluded due to not being adolescents (i.e. ≥ 20 years). Although one of our specified inclusion criteria was to be 13–18 years, we decided to retain the fourteen 19-year-old participants, due to them still being classed as adolescents and to maintain statistical power. This resulted in a final sample size of ninety-one participants. Eight participants elected to not be weighed and/or measured; therefore, BMI z-score and weight category data are missing for these participants. Six participants violated parameters on the Stop Signal task^(36)^, and therefore, their data were entered as missing for the SSRT variable. The sample was largely representative of the UK youth population in terms of ethnicity and weight status but not gender^(40–42)^.

**Table 1.** Sample characteristics, split by condition

	Experimental condition (*n* 47)		Control condition (*n* 44)		Overall (*n* 91)	
	Mean	sd	Mean	sd	Mean	sd
Age (years)	17·78	1·54	17·83	1·44	17·83	1·48
Gender (female: male: non-binary)	37: 9: 1 (78·72 % female)		26: 17: 1 (59·09 % female)		63: 26: 2 (69·23 % female)	
Ethnicity (White: Asian: Black: Arab: Mixed)	38: 4: 2: 1: 2 (80·85 % white)		36: 4: 3: 1: 0 (81·81 % white)		74: 8: 5: 2: 2 (81·3 % white)	
IMD decile	4·54	2·30	4·00	2·65	4·30 (78 % missing)	2·41
Time spent on VGLSP per week (hours)	5·11	6·76	7·54	8·65	6·28	7·78
Previously used VGLSP (yes: no)	30: 17 (63·83 % yes)		34: 10 (77·3 % yes)		64: 27 (70·33 % yes)	
BMI z score*	0·62	1·03	0·45	1·16	0·54	1·09
Weight category (Thinness: Healthy: Overweight: Obesity: Missing)**	0: 29: 12: 4: 2 (61·70 % healthy weight)		0: 30: 5: 3: 6 (68·18 % healthy weight)		0: 59: 17: 7: 8(64·84 % healthy weight)	
Test brand (Doritos) liking (100 mm VAS)	64·64	26·70	60·36·	19·68	62·57	23·54
Hunger (100 m VAS)	44·85	19·79	45·59·	22·63	45·21	21·10
Streamer (Ninja) familiarity (100 mm VAS)	34·60	28·84	41·77	34·15	38·07	31·55
Videogame (Fortnite) familiarity (100 mm VAS)	68·94	26·51	71·07	22·72	69·97	24·63
Stream liking (100 mm VAS)	42·74	20·94	44·07	23·35	43·38	22·02
Doritos consumed (kcal)	53·77	36·97	53·41	43·55	53·60	40·1
Overall crisps consumed (kcal)	105·43	67·15	109·85	71·79	107·56	69·08
Gaze duration (seconds)	5·75	4·86	3·24	2·99	4·54	4·24
SSRT (milliseconds)	10·19	48·32	9·93	53·16	10·06	50·47
Advert correctly recalled (Yes: No)	45: 2 (95·74 % yes)		35: 9 (79·55 % yes)		80: 11 (87·91 % yes)	
Aim correctly identified (Yes: No)	12: 35(74·47 % no)		1: 43 (97·73 % no)		13: 78 (85·71 % no)	

IMD, index of multiple deprivation; VGLSP, video game live streaming platform; VAS, visual analogue rating scale; SSRT, stop signal reaction time.

*A standardised BMI z score based on age was calculated using WHO reference data^(37)^. **Weight category cut-offs were defined as thinness: < –2 sd, healthy weight: > –2 sd < +1 sd, overweight: > +1 sd, obesity: > +2 sd^(37)^.

To examine which variables should be included as covariates in the main analyses, Pearson’s correlations were calculated. As shown in Table 2, stream liking and weekly time spent on VGLSP were positively correlated with Doritos intake. Familiarity with the video game Fortnite was positively correlated with overall intake. Welch’s two sample *t* tests were calculated for categorical variables (gender [m/f ], advert recall [y/n] and aim guess [y/n]). There was a significant difference in overall intake based on gender, with males consuming significantly more than females (*P* < 0·05). There were no other significant differences in Doritos or overall intake (all *P* > 0·05).

**Table 2.** Pearson’s correlations between dependent variables and covariates

	Doritos intake (kcal)	Overall intake (kcal)
Hunger (VAS mm)	0·15	0·04
BMIz	0·09	0·07
Age (years)	−0·04	−0·11
IMD decile	0·00	0·05
Streamer familiarity (VAS mm)	0·10	0·14
Videogame familiarity (VAS mm)	0·16	0·22^*^
Stream liking (VAS mm)	0·21^*^	0·13
Doritos liking (VAS mm)	0·03	0·03
Time spent on VGLSP per week (h)	0·24^*^	0·16

IMD, index of multiple deprivation; VGLSP, video game live streaming platform; VAS, visual analogue rating scale.

* *P* < 0·05.

### Model 1: Predictors of Doritos intake

Examination of the relevant plots revealed one problematic (influential) case. This case was removed, resulting in the assumptions of linearity and homoscedasticity being met. Assumptions of normality were also met. Multicollinearity (some VIF > 5) was resolved by mean-centering the gaze duration and SSRT variables (all VIF < 3). The first linear model included Doritos intake as the dependent variable. VGLSP weekly use and stream liking were included as covariates due to their significant correlation with Doritos intake. The inclusion of two additional covariates did not impact the required sample size (*n* 90, G * Power).

The overall model was non-significant (R^2^ = 0·16, F(9, 74) = 1·55, *P* = 0·15). As shown in Table 3, step one of the hierarchical model significantly predicted approximately 15 % of variance in Doritos intake. The only significant predictor was weekly VGLSP use, with greater time spent using VGLSP per week being associated with increased Doritos intake. The size of this effect was medium-large^(43)^.

**Table 3.** Model 1, predictors of Doritos intake

	Cumulative		Simultaneous			
	R^2^ change	F- change	B	se	CI	*P*, Cohen’s f
Step one	**0·15**	(5, 78) 2·75^*^				
Condition			−02·03	10·74	−21·16, 17·08	0·83, 0·03
Gaze duration			0·26	1·37	−2·46, 2·99	0·85, 0·02
SSRT			−0·12	0·14	−0·39, 0·16	0·39, 0·06
Stream liking			0·38	0·22	−0·06, 0·81	0·93, 0·28
VGLSP total			**1·78**	0·74^*^	**0·31, 3·25**	**0·02, 0·31**
Step two	0·01	(4, 74) 0·20				
Condition*gaze duration			−1·07	2·53	−6·10, 3·97	0·67, 0·03
Condition*SSRT			0·10	0·19	−0·27, 0·47	0·58, 0·05
Gaze duration*SSRT			0·02	0·30	−0·0, 0·08	0·55, 0·03
Condition*gaze duration*SSRT			−0·03	0·05	−0·13, 0·07	0·50, 0·08

VGLSP, video game live streaming platform; SSRT, stop signal reaction time.

* *P* < 0·05 (also highlighted in bold).

### Model 2: predictors of overall snack intake

Examination of the relevant plots and tests revealed that assumptions of normality, linearity and homoscedasticity were met. Again, multicollinearity (some VIF > 5) was resolved by mean-centring the gaze duration and SSRT variables (all VIF < 3). The second linear model included overall snack intake as the dependent variable. Fortnite familiarity was included as a covariate due to its significant correlation with overall snack intake, and gender was included as males had significantly greater overall snack intake than females.

The overall model was non-significant (R^2^ = 0·14, F(9, 73) = 1·29, *P* = 0·26. As shown in Table 4, both steps in the model were also non-significant. The only significant predictor was gender, with being male associated with greater overall intake. The size of this effect was medium-large^(43)^.

**Table 4.** Model 2, predictors of overall snack intake

	Cumulative		Simultaneous			
	R^2^ change	*F*-change	B	se	CI	*P*, Cohen’s *f*
Step one	0·10	(5, 77) 1·73				
Condition			4·51	17·05	−29·46, 38·49	0·79, 0·07
Gaze duration			1·77	2·44	−3·09, 6·62	0·47, 0·08
SSRT			−0·18	0·25	−0·67, 0·32	0·48, 0·05
Gender			**−44·30**	20·39^*^	**−84·93, –3·67**	**0·03, 0·22**
Fortnite familiarity			0·34·	0·35	−0·35, 1·04	0·32, 0·23
Step two	0·04	(4, 73) 0·76				
Condition*gaze duration			−0·97	4·53	−10·00, 8·06	0·83, 0·01
Condition*SSRT			0·12	0·33	−0·53, 0·77	0·71, 0·04
Gaze duration*SSRT			0·02	0·53	−0·08, 0·13	0·68, 0·07
Condition*gaze duration*SSRT			−0·14	0·09	−0·31, 0·03	0·12, 0·19

SSRT, stop signal reaction time.

* *P* < 0·05 (also highlighted in bold).

### Sensitivity analyses

Neither the removal of participants aged 19 years and older nor the removal of aim guessers notably impacted any models or individual predictors. See Appendix for full sensitivity analyses.

## Discussion

This between-subjects randomised controlled trial assessed the effect of HFSS food marketing exposure (compared to non-food marketing exposure) in a mock Twitch stream on immediate marketed and overall snack intake. It was firstly hypothesised that participants in the food marketing condition would have greater marketed and overall snack intake than those in the non-food marketing condition. No significant association between the condition and either marketed or overall snack intake was found, and therefore, this hypothesis is rejected. However, although the overall model was non-significant, it was found that greater weekly use of VGLSP was associated with increased marketed snack intake. It was secondly hypothesised that participants who showed greater attentional bias towards the food advert would have greater marketed and overall snack intake, and those with poorer inhibitory control would also have greater marketed and overall snack intake in response to the food advert. No significant interaction between the condition and either gaze duration or inhibitory control in terms of predicting marketed or overall snack intake was found. Therefore, both hypotheses are unsupported. Finally, it was hypothesised that participants with the highest rates of attentional bias *and* the poorest inhibitory control would have greater marketed and overall snack intake in response to food marketing. No significant interaction effect of gaze duration and inhibitory control in response to food marketing was found, and therefore, this hypothesis is also unsupported.

The finding that acute exposure to Twitch/VGLSP-based food marketing was not significantly associated with immediate snack intake is largely inconsistent with existing research. Several reviews have identified an association between food marketing exposure via both traditional media (e.g. TV) and digital media (advergames, social media [including influencers]) and greater HFSS food intake^(10,11,15)^. One meta-analysis found that experimental exposure to key marketing techniques used on VGLSP (influencer or digital game-based marketing) led to the consumption of an additional 37 kcal in HFSS food^(15)^. In comparison, a negligible difference in HFSS snack consumption between the experimental and control conditions (< 5 kcal) was found in this study. While we sought to examine the impact of an in-stream static banner advert specifically, it is important to note that food marketing on Twitch is often synergistic and includes a range of different in-stream advert types, such as jersey patches, product placements, banners and video adverts^(9)^. Therefore, the behavioural impact of exposure to food marketing on Twitch may be underestimated in this study.

In addition to this, while experimentally impractical to simulate, a core Twitch viewer spends nearly 5 h every day on the platform^(44)^. This greater duration of, and more sustained, exposure is likely to have more of an impact on eating behaviours (i.e. a dose-response relationship^(45)^). Indeed, in the current study, it was found that, although the overall model was non-significant, higher weekly use of VGLSP was associated with greater marketed snack intake. This complements findings that higher recall of food marketing on VGLSP is associated with greater HFSS food consumption in both adolescents and adults^(13,14)^. The test brand used in the current study (Doritos) has strong ties with streaming and gaming, and therefore, it is likely that a more frequent Twitch (or VGLSP in general) user would have greater habitual exposure to Doritos marketing. Doritos is the most frequently marketed and mentioned (i.e. in the chatroom) snack food brand on Twitch, and the brand has its own ‘chip’ emote which can be used in the chatroom^(3,9)^. However, further experimental research is needed to isolate this effect and determine whether it can be replicated.

The finding that attentional bias towards the food advert was not associated with intake is also inconsistent with existing research. Previous studies found that children with a higher gaze duration for food cues in an advergame ate more of the marketed HFSS snacks^(22)^. It is possible that differences were driven by variation in (i) the prominence of the advert (i.e. size, positioning) and/or (ii) the involvement of the media (i.e. a pairs advergame *v*. a Fortnite stream). Indeed, more prominent adverts tend to be remembered better, and prominence and involvement are known to interact in in-game advertising (IGA; i.e. at moderate involvement prominent brands are recognised better than peripheral brands)^(46)^. It may be that more prominent and dynamic adverts (e.g. looping banner adverts, interactive product placement) are required to capture attention when watching an involved Twitch stream.

Similarly, the finding that inhibitory control was not associated with intake is largely in disagreement with previous research. In one study, children whose inhibitory control was strengthened (in relation to the advertised food, using a go-no-go food task) were found to consume significantly fewer calories after playing an advergame promoting energy-dense foods, relative to those who did not complete the task^(26)^. In contrast, in this study, having poorer inhibitory control for commonly advertised branded foods on Twitch was not significantly associated with greater branded or overall snack consumption in response to the food advert. It may be that because our sample was predominantly made up of older adolescents, psychological mechanisms behind inhibitory control were more developed, leading to a diminished effect^(16)^. Indeed, a meta-analysis of the effects of exposure to food-related cues on inhibitory control in adults found no effect, suggesting that age is important^(47)^.

Overall, our findings are not consistent with the dual process model of cognition, which predicts that both increased attentional bias towards food-related cues and poor food-related inhibitory control are likely to be associated with increased cue-induced food consumption. It is possible that our sample, in general, did not have an attentional bias toward food cues and/or poor food-related inhibitory control. It may also be that this model is not applicable to food marketing effects in the context of videogame livestreaming. VGLSP use a unique range of marketing integration strategies (e.g. saturation, congruency, social influence) which may interact differently with measures of attentional bias and inhibitory control^(48)^. However, further research examining responses to various advert formats in VGLSP is required to test this assertion.

The current study has some limitations. Lightly Salted Doritos were served to participants without an accompanying dip. This may not be representative of the typical eating experience and therefore could have resulted in lower intake. Owing to difficulties recruiting the target age group, opportunity sampling was used, which meant that the sample was predominantly female, which is likely not representative of the typical Twitch user (approximately 70 % are male^(49)^), and there were more females in the experimental condition. Similarly, the mean time spent on VGLSP per week was approximately 2 hours less in the experimental condition, relative to the control condition. It was found that being male and spending more time on VGLSP were significantly associated with greater overall snack and Doritos intake, respectively, so this may have biased results towards the null. Future studies may wish to use quota sampling to ensure balanced assignment to conditions based on gender and VGLSP use.

It is recommended that future studies assess the effect of other commonly used advertising placements on Twitch and other VGLSP both in isolation and together (i.e. their combined effect on eating behaviour). In addition, it would be beneficial to replicate the study in different samples, such as younger adolescents, males and those who use VGLSP regularly (i.e. weekly), as these subgroups are likely to be most impacted by advertising on VGLSP. Future research should also endeavour to investigate the impact of exposure to food marketing via VGLSP on food intake over time (e.g. using screen recording software to monitor exposure) and on other food-related outcomes such as norms, attitudes and intended purchase.

### Conclusion

Overall, findings suggest that exposure to a static food banner advert in a mock Twitch stream was not significantly associated with immediate marketed or overall snack intake in adolescents. Moreover, attentional bias and inhibitory control did not appear to have any significant impact on consumption in response to food marketing. However, although the overall model was non-significant, we did find that higher weekly use of VGLSP was associated with greater marketed snack intake. The findings are largely inconsistent with existing research exploring the impacts of food marketing exposure via digital media on food intake. Future research is recommended to explore the impacts of different advertising placements on VGLSP (and their combined effect), replication in specific samples and assessment of the impact of longer-term exposure to this marketing.

## Supporting information

Evans et al. supplementary material Evans et al. supplementary material

## Supplementary material

For supplementary material accompanying this paper visit https://doi.org/10.1017/S1368980025100487

## References

[1] WHO (2016) Report of the Commission on Ending Childhood Obesity. https://www.who.int/publications/i/item/9789241510066 (accessed July 2024).

[2] WHO (2016) Tackling Food Marketing to Children in a Digital World: Trans-Disciplinary Perspectives. https://www.euro.who.int/__data/assets/pdf_file/0017/322226/Tackling-food-marketing-children-digital-world-trans-disciplinary-perspectives-en.pdf (accessed July 2024).

[3] Edwards CG , Pollack CC , Pritschet SJ et al. (2022) Prevalence and comparisons of alcohol, candy, energy drink, snack, soda, and restaurant brand and product marketing on Twitch, Facebook Gaming and YouTube Gaming. Public Health Nutr 25, 1–12. 10.1017/S1368980021004420 PMC8593406 34693900

[4] Evans R , Christiansen P , Masterson T et al. (2024) Food and non-alcoholic beverage marketing via Fortnite streamers on Twitch: a content analysis. Appetite 195, 107207. 38218416 10.1016/j.appet.2024.107207

[5] Hatchet S (2023) Video Game Streaming Trends Report 2022 Yearly Report. https://streamhatchet.com/blog/blog-2022-yearly-video-game-live-streaming-trends-report/ (accessed July 2024).

[6] May (2021) Streamlabs and Stream Hatchet Q4 Live Streaming Industry Report. https://streamlabs.com/content-hub/post/streamlabs-and-stream-hatchet-q4-live-streaming-industry-report (accessed July 2024).

[7] Ofcom (2023) Children and Parents: Media Use and Attitudes Report 2023. https://www.ofcom.org.uk/media-use-and-attitudes/media-habits-children/children-and-parents-media-use-and-attitudes-report-2023 (accessed July 2024).

[8] Aiello (2021) Nickelodeon Kids’ Choice Awards 2021 Nominations: See the Complete List. https://www.eonline.com/uk/news/1233568/nickelodeon-kids-choice-awards-2021-nominations-see-the-complete-list (accessed July 2024).

[9] Brooks A (2022) Brands in Video Gaming and Esports Report. https://streamhatchet.com/blog/blog-brands-in-video-gaming-and-esports-report/(accessed July 2024).

[10] Boyland E , McGale L , Maden M et al. (2022) Association of food and nonalcoholic beverage marketing with children and adolescents’ eating behaviors and health: a systematic review and meta-analysis. JAMA Pediatr 176, e221037. 35499839 10.1001/jamapediatrics.2022.1037 PMC9062773

[11] Packer J , Russell SJ , Siovolgyi G et al. (2022) The impact on dietary outcomes of celebrities and influencers in marketing unhealthy foods to children: a systematic review and meta-analysis. Nutrients 14, 434. 35276800 10.3390/nu14030434 PMC8837952

[12] Russell SJ , Croker H & Viner RM (2019) The effect of screen advertising on children’s dietary intake: a systematic review and meta-analysis. Obes Rev 20, 554–568. 30576057 10.1111/obr.12812 PMC6446725

[13] Evans R , Christiansen P , Masterson T et al. (2023) Recall of food marketing on videogame livestreaming platforms: associations with adolescent diet-related behaviours and health. Appetite 186, 106584. 37127245 10.1016/j.appet.2023.106584

[14] Pollack CC , Gilbert-Diamond D , Emond JA et al. (2021) Twitch user perceptions, attitudes and behaviours in relation to food and beverage marketing on Twitch compared with YouTube. J Nutr Sci 10, e32. 34094513 10.1017/jns.2021.22 PMC8141682

[15] Evans RK , Christiansen P , Finlay A et al. (2023) A systematic review and meta-analysis of the effect of digital game-based or influencer food and non-alcoholic beverage marketing on children and adolescents: exploring hierarchy of effects outcomes. Obes Rev 24, e13630. 37608618 10.1111/obr.13630

[16] Harris JL , Yokum S & Fleming-Milici F (2021) Hooked on junk: emerging evidence on how food marketing affects adolescents’ diets and long-term health. Curr Addict Rep 8, 19–27.

[17] Elliott C , Truman E & Stephenson N (2022) Food marketing and power: teen-identified indicators of targeted food marketing. Int J Environ Res Public Health 19, 7815. 35805473 10.3390/ijerph19137815 PMC9265287

[18] Kakoschke N , Kemps E & Tiggemann M (2015) Combined effects of cognitive bias for food cues and poor inhibitory control on unhealthy food intake. Appetite 87, 358–364. 25592403 10.1016/j.appet.2015.01.004

[19] Harris JL & Bargh JA (2009) Television viewing and unhealthy diet: implications for children and media interventions. Health Commun 24, 660–673. 20183373 10.1080/10410230903242267 PMC2829711

[20] Harris JL , Bargh JA & Brownell KD (2009) Priming effects of television food advertising on eating behavior. Health Psychol 28, 404. 19594263 10.1037/a0014399 PMC2743554

[21] Hardman CA , Jones A , Burton S et al. (2021) Food-related attentional bias and its associations with appetitive motivation and body weight: a systematic review and meta-analysis. Appetite 157, 104986. 33039507 10.1016/j.appet.2020.104986

[22] Folkvord F , Anschutz DJ , Wiers RW et al. (2015) The role of attentional bias in the effect of food advertising on actual food intake among children. Appetite 84, 251–258. 25451582 10.1016/j.appet.2014.10.016

[23] Pollack CC , Emond JA & Masterson TD (2022) Associations between adolescent and young adult External Food Cue Responsiveness (EFCR) and brand recall, product craving and product purchasing in the livestreaming food marketing environment. Public Health Nutr 25, 3036–3043. 35920082 10.1017/S1368980022001628 PMC9991748

[24] Meng X , Huang D , Ao H et al. (2020) Food cue recruits increased reward processing and decreased inhibitory control processing in the obese/overweight: an activation likelihood estimation meta-analysis of fMRI studies. Obes Res Clin Pract 14, 127–135. 32098756 10.1016/j.orcp.2020.02.004

[25] McGreen J , Kemps E & Tiggemann M (2023) The relationship between inhibitory control and food consumption or choice: a systematic review and meta-analysis. Appetite 183, 106466. 36690185 10.1016/j.appet.2023.106466

[26] Folkvord F , Veling H & Hoeken H (2016) Targeting implicit approach reactions to snack food in children: effects on intake. Health Psychol 35, 919. 27505216 10.1037/hea0000365

[27] Yeum D , Jimenez CA , Emond JA et al. (2023) Differential neural reward reactivity in response to food advertising medium in children. Front Neurosci 17, 1052384. 36816130 10.3389/fnins.2023.1052384 PMC9933514

[28] Coates AE , Hardman CA , Halford JCG et al. (2019) The effect of influencer marketing of food and a ‘protective’ advertising disclosure on children’s food intake. Pediatr Obes 14, e12540. 31168959 10.1111/ijpo.12540

[29] Sommet N , Weissman DL , Cheutin N et al. (2023) How many participants do I need to test an interaction? Conducting an appropriate power analysis and achieving sufficient power to detect an interaction. Adv Methods Pract Psychol Science 6, 25152459231178728.

[30] Conway J (2023) Leading Snack Brands Ranked by Sales Value in the United Kingdom (UK) 2017. https://www.statista.com/statistics/788123/leading-snack-brands-ranked-by-sales-value-in-the-united-kingdom/ (accessed July 2024).

[31] Cameron I (2022) Adidas UK Sales Rise in FY21 Results: Charged. https://www.chargedretail.co.uk/2022/10/07/adidas-uk-sales-rise-in-fy21-results/ (accessed July 2024).

[32] Robinson E , Kersbergen I , Brunstrom JM et al. (2014) I’m watching you. Awareness that food consumption is being monitored is a demand characteristic in eating-behaviour experiments. Appetite 83, 19–25. 25086209 10.1016/j.appet.2014.07.029

[33] Logan GD , Cowan WB & Davis KA (1984) On the ability to inhibit simple and choice reaction time responses: a model and a method. J Exp Psychol: Hum Percept Perform 10, 276. 6232345 10.1037//0096-1523.10.2.276

[34] Chao AM , Fogelman N , Hart R et al. (2020) A laboratory-based study of the priming effects of food cues and stress on hunger and food intake in individuals with obesity. Obesity 28, 2090–2097. 32918391 10.1002/oby.22952 PMC7644599

[35] Kay E , Kemps E , Prichard I et al. (2023) Instagram-based priming to nudge drink choices: subtlety is not the answer. Appetite 180, 106337. 36210015 10.1016/j.appet.2022.106337

[36] Verbruggen F , Aron AR , Band GP et al. (2019) A consensus guide to capturing the ability to inhibit actions and impulsive behaviors in the stop-signal task. elife 8, e46323. 31033438 10.7554/eLife.46323 PMC6533084

[37] WHO (2020) Growth Reference 5–19 Years. BMI-for-Age (5–19 Years). https://www.who.int/growthref/who2007_bmi_for_age/en/ (accessed July 2024).

[38] Freedman DS , Lawman HG , Skinner AC et al. (2015) Validity of the WHO cutoffs for biologically implausible values of weight, height, BMI in children, adolescents in NHANES from 1999 through 2012. Am J Clin Nutr 102, 1000–1006. 26377160 10.3945/ajcn.115.115576 PMC4631693

[39] James G (2013) An Introduction to Statistical Learning. New York: Springer.

[40] ONS (2018) Young People by Ethnicity in England and UK. https://www.ons.gov.uk/peoplepopulationandcommunity/culturalidentity/ethnicity/adhocs/008436youngpeoplebyethnicityinenglandanduk (accessed March 2025).

[41] ONS (2019) Youth Age Groups - Breakdown of Age Groups 13 to 29 Years and 16 to 29 Years by Sex, Regions and Education Status in UK. https://www.ons.gov.uk/peoplepopulationandcommunity/populationandmigration/populationestimates/adhocs/010127youthagegroupsbreakdownofagegroups13to29yearsand16to29yearsbysexregionsandeducationstatusinuk (accessed March 2025).

[42] Stiebahl S (2025) Obesity Statistics. London: House of Commons Library.

[43] Cohen J (1988) Statistical Power Analysis for the Behavioral Sciences. Hillsdale, NJ: Lawrence Erlbaum Associates.

[44] Hatchet S (2022) Video Game Streaming Trends Report 2022 First Quarter Report. https://streamhatchet.com/blog/video-game-live-streaming-trends-q1-2022-report/ (accessed July 2024).

[45] Norman J , Kelly B , Boyland E et al. (2016) The impact of marketing and advertising on food behaviours: evaluating the evidence for a causal relationship. Curr Nutr Reports 5, 139–149.

[46] Terlutter R & Capella ML (2013) The gamification of advertising: analysis and research directions of in-game advertising, advergames, and advertising in social network games. J Advertising 42, 95–112.

[47] Jones A , Robinson E , Duckworth J et al. (2018) The effects of exposure to appetitive cues on inhibitory control: a meta-analytic investigation. Appetite 128, 271–282. 29935289 10.1016/j.appet.2018.06.024

[48] Maksi S , Keller K , Dardis F et al. (2024) The Food and Beverage Cues in Digital Marketing (FBCDM) Model: special considerations of social media, gaming, and livestreaming environments for food marketing and eating behavior research. Front Nutr 10, 1325265. 38384857 10.3389/fnut.2023.1325265 PMC10880034

[49] Statista (2024) Distribution of Twitch.tv Users Worldwide as of November 2024, by Gender 2024. https://www.statista.com/statistics/633937/twitch-user-gender-worldwide/ (accessed March 2025).

