# Supplementary material for: The impact of food marketing via video game live streaming on snack intake in adolescents: a randomised controlled trial
Source: Public Health Nutr. 2025 Jun 3;28(1):e109. doi: 10.1017/S1368980025100487 (PMC12305382; doi:10.1017/S1368980025100487)

**Appendix**

*Mock Twitch video stimuli*

**Figure A1**: Food banner advert


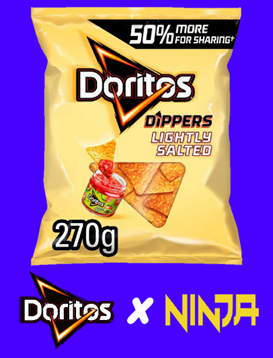


**Figure A2**: Non-food banner advert


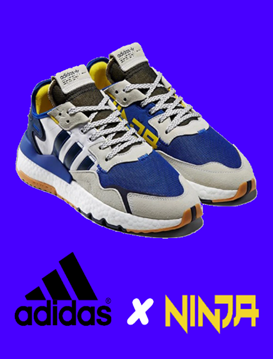


**Figure A3**: Still from mock Twitch video


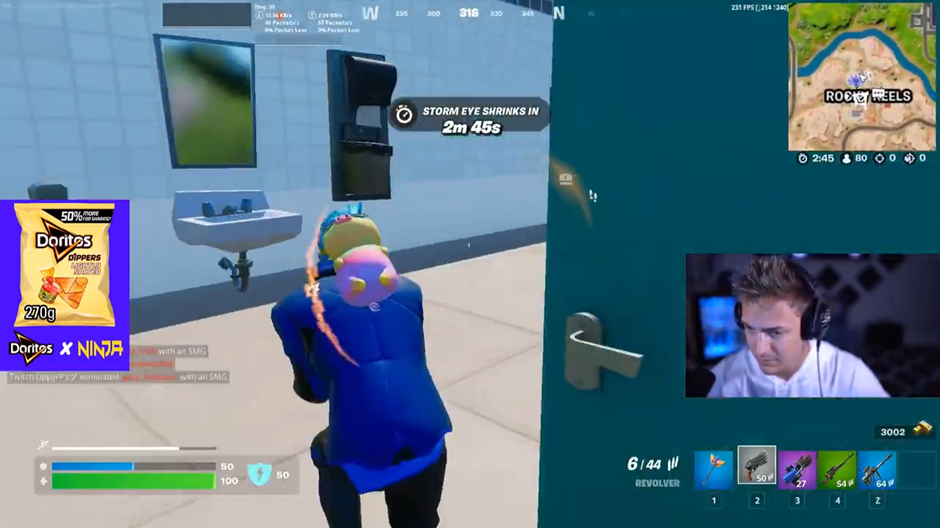


Video with food marketing - [https://www.youtube.com/watch?v=1veGP2rV4q4&feature=youtu.be](https://url.avanan.click/v2/r02/___https://www.youtube.com/watch?v=1veGP2rV4q4&feature=youtu.be___.YXAxZTpjYW1icmlkZ2Vvcmc6YTpvOmQ2YTA3ZWZkMTkwZTU1YjI0NmUwYjQ1NDEwYjcxOTdmOjc6NzBhNDpkZmIzOGIzZTRiNjNkYzRjMmUzYjA4M2EwYjhiY2JkZTgzNzk3ZGU5NTc1ODEzOTg3OWZkM2Y2YjQ5MmU1NTlkOnA6VDpG)

Video with non-food marketing - [https://www.youtube.com/watch?v=DOv86UNVHeY](https://url.avanan.click/v2/r02/___https://www.youtube.com/watch?v=DOv86UNVHeY___.YXAxZTpjYW1icmlkZ2Vvcmc6YTpvOmQ2YTA3ZWZkMTkwZTU1YjI0NmUwYjQ1NDEwYjcxOTdmOjc6NWRkZjo3ZTliY2FhY2E2ZWU2YWY3YWQ0NGUzZTg5OTA5ZTIwNmU5MmRhZWI3N2FjY2U3MThjMTFmZWU0NDQwNjI1NDVhOnA6VDpG)

*Stop Signal Task stimuli*

**Figure A4**: Images used in the Stop Signal Task


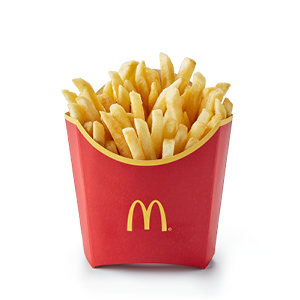

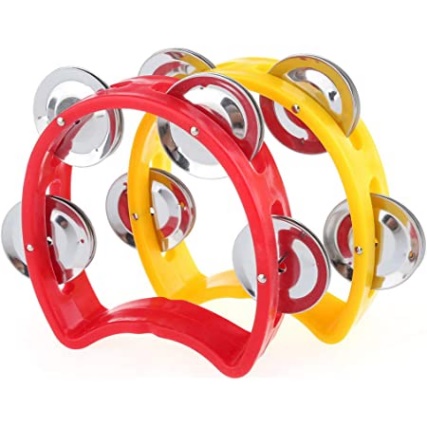


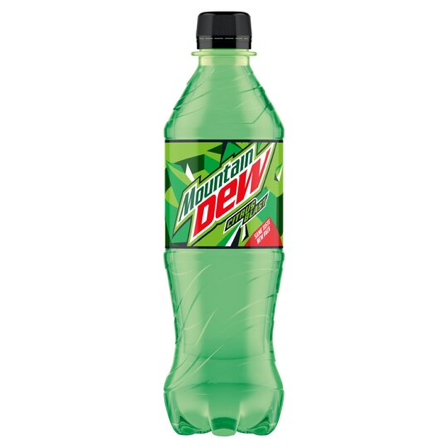

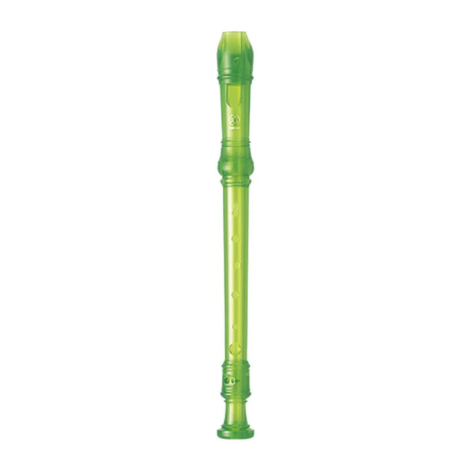


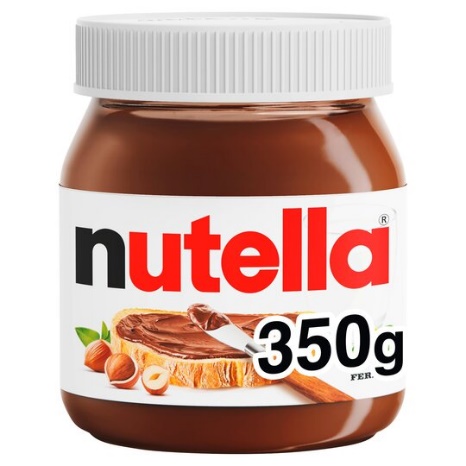

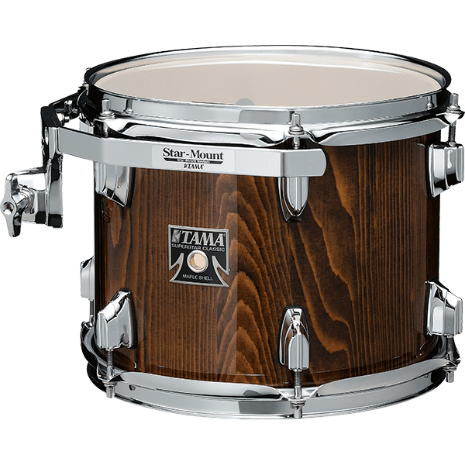


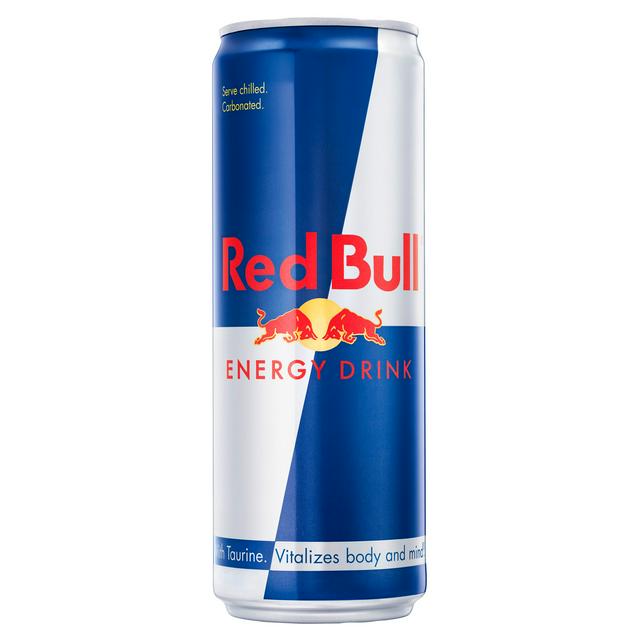

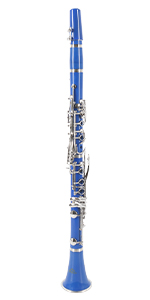


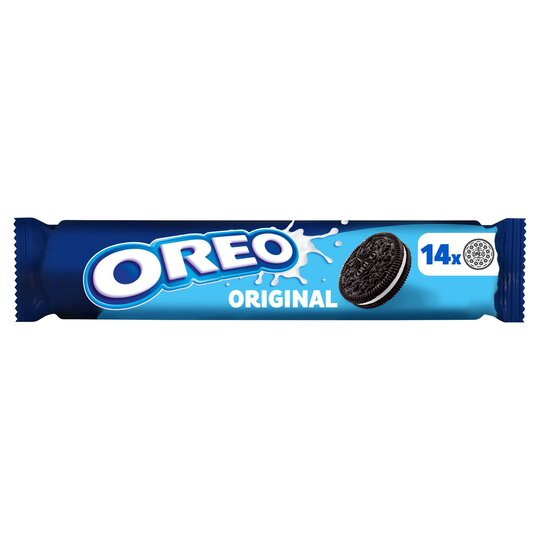

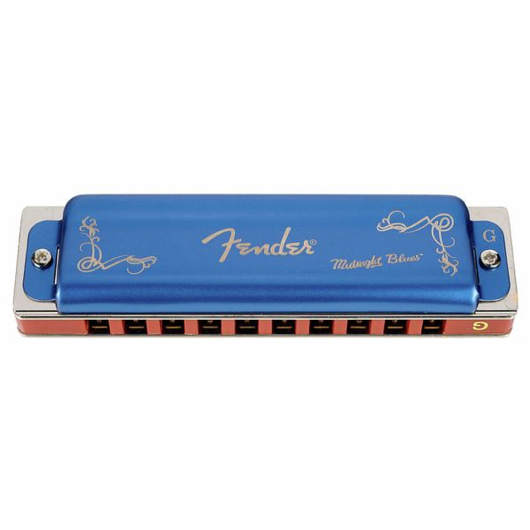


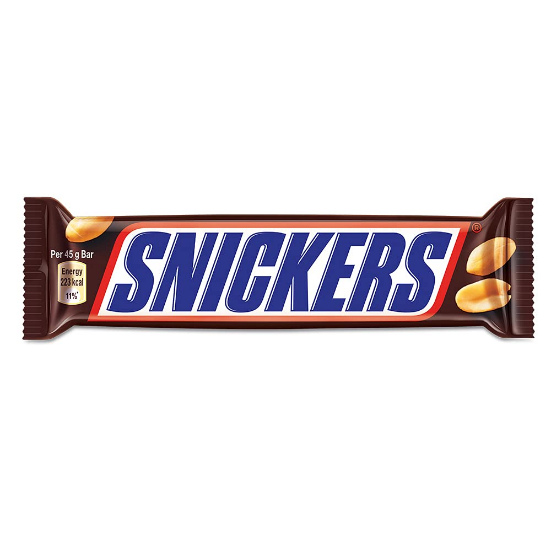

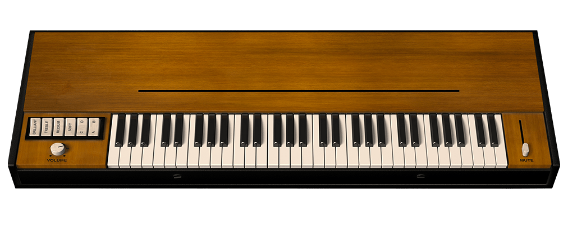


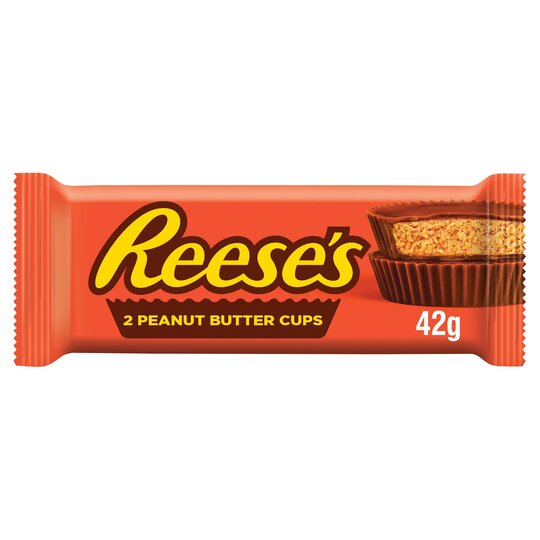

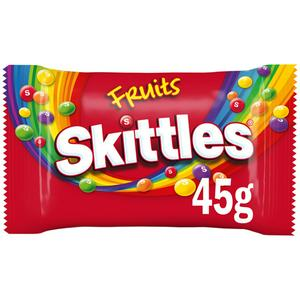

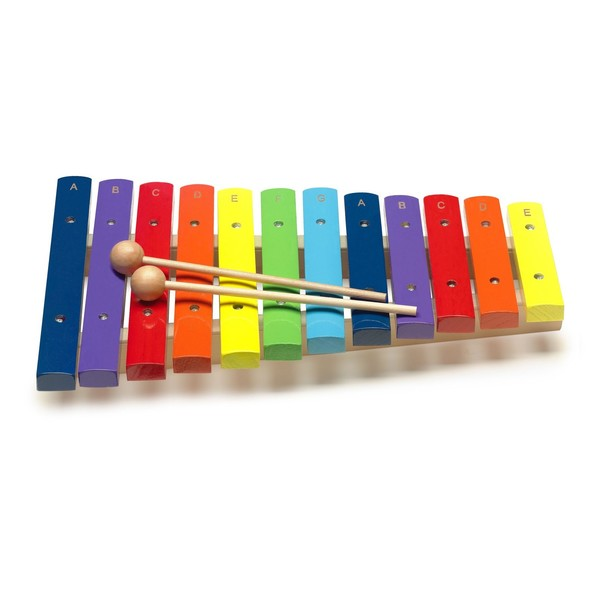


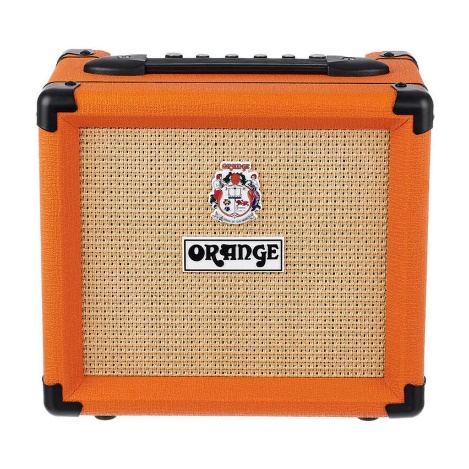


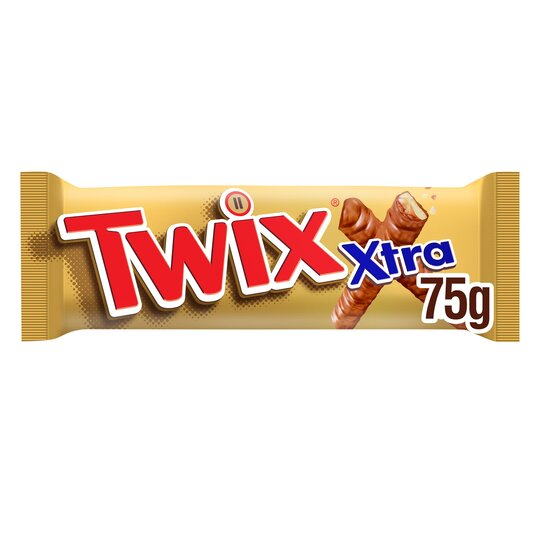


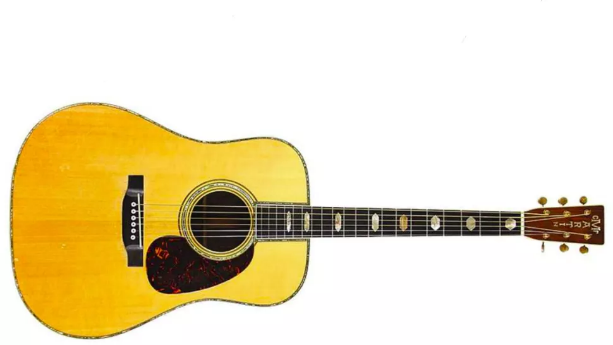


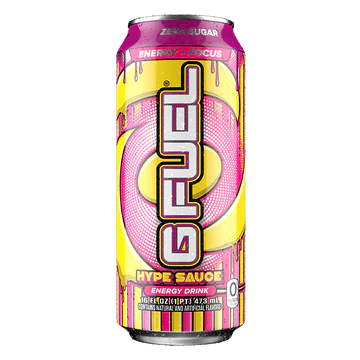

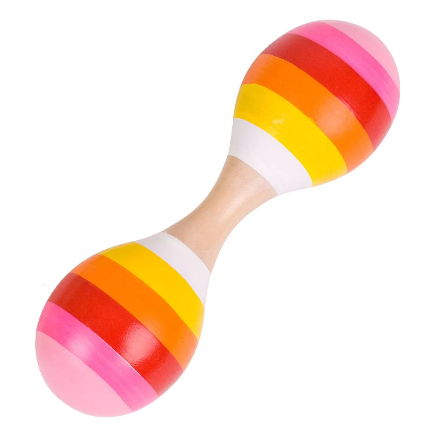


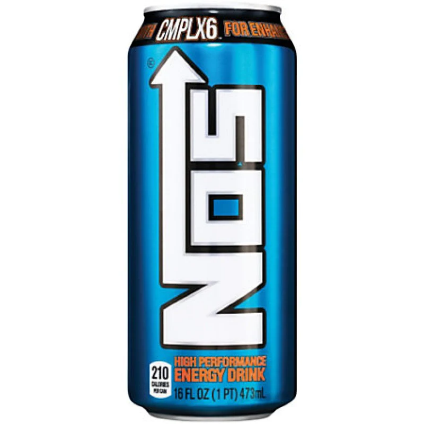

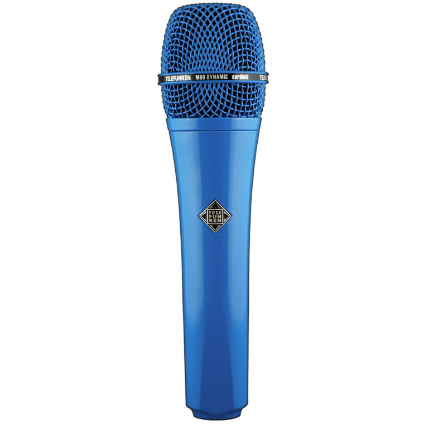


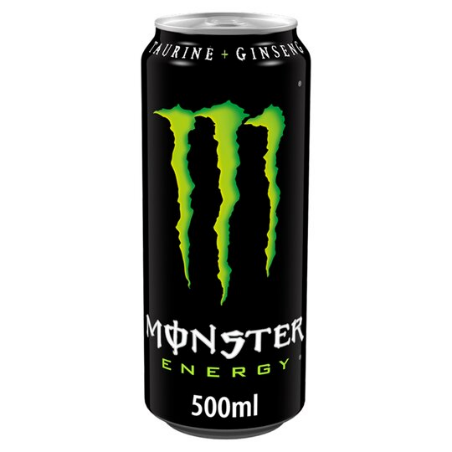

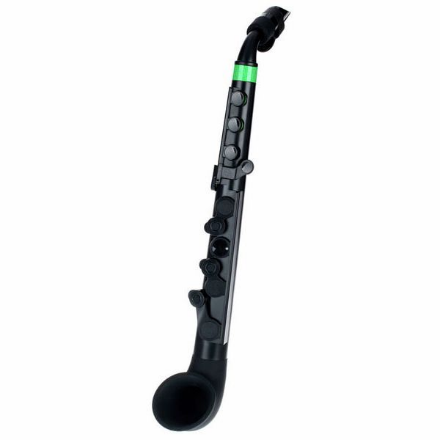


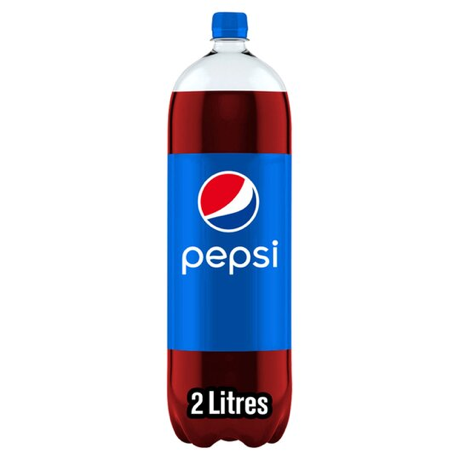

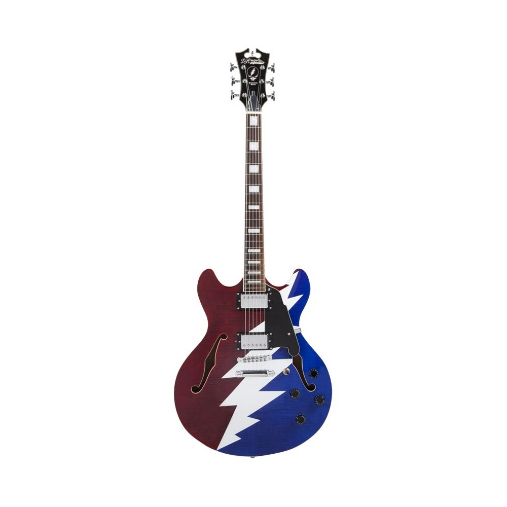


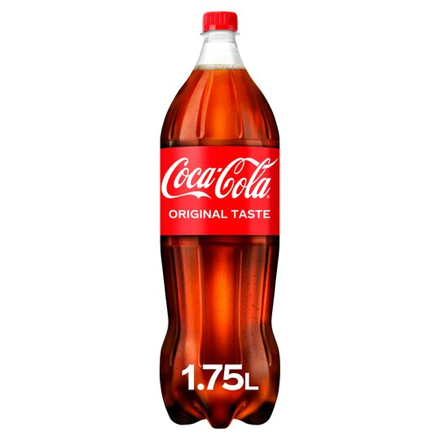

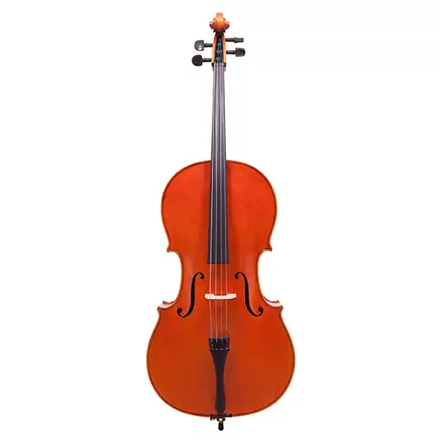


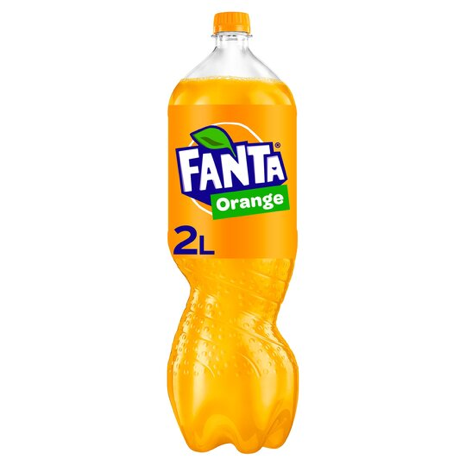

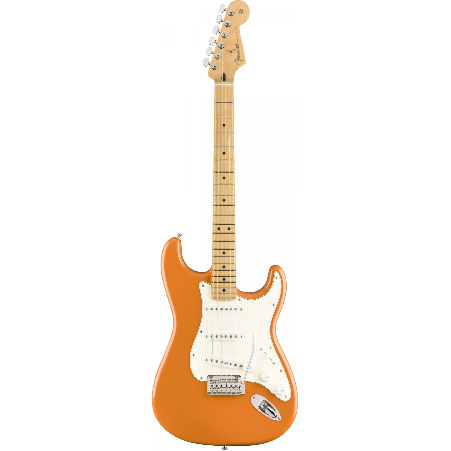


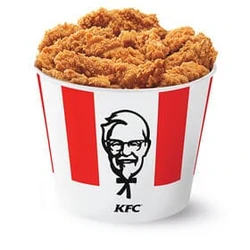

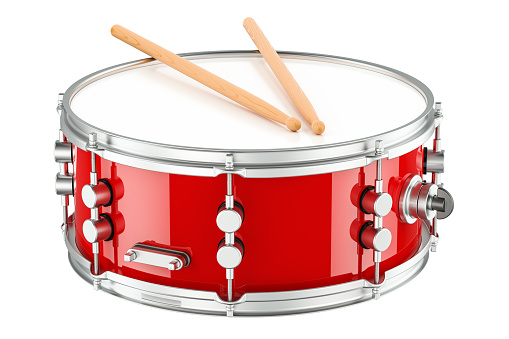


*Experimental set-up*

**Figure A5**: Eye tracker set-up


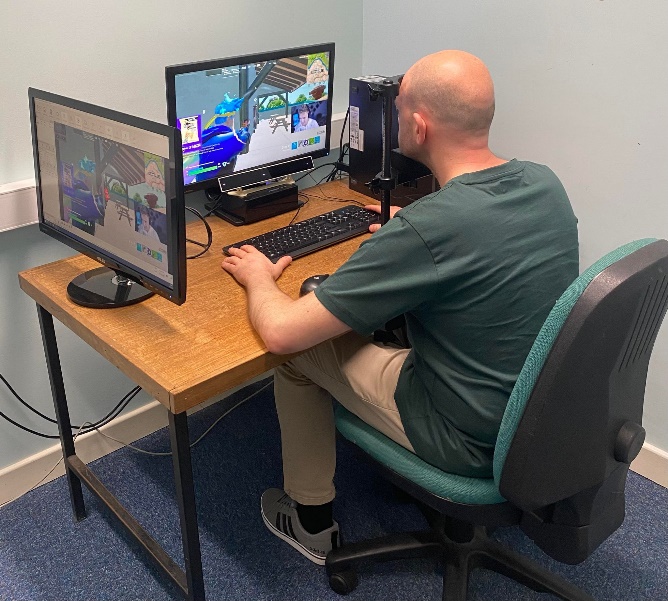

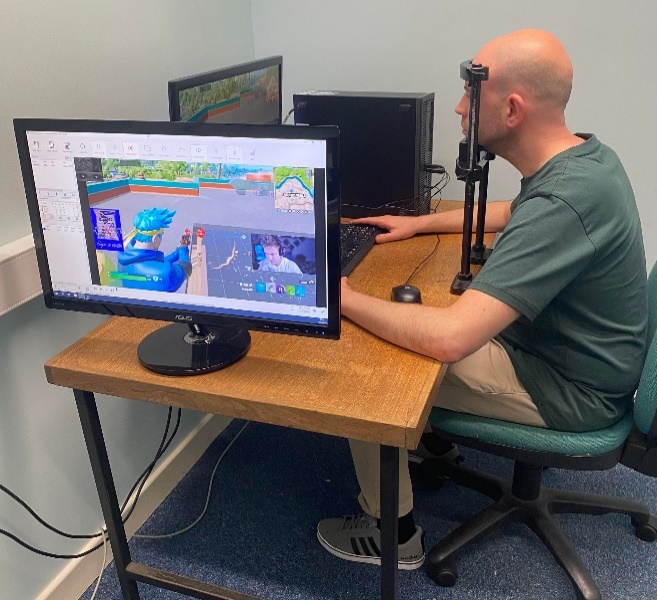


**Figure A6**: Test food presentation


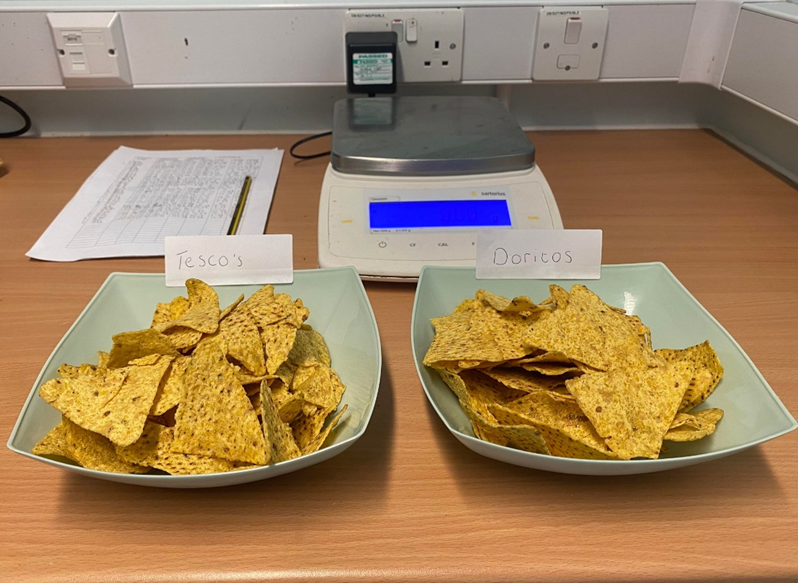


*Sensitivity analyses*

**Table A1**: Model 1, predictors of Doritos intake (with participants aged 19 and over excluded)

|  | Cumulative | | Simultaneous | |  |
| --- | --- | --- | --- | --- | --- |
|  | R^2^ change | F- change | B (SE) | CIs | *p*, Cohen’s *f* |
| **Step one** | .17 | **(5, 65) 2.58*** |  |  |  |
| Condition |  |  | -4.10 (10.42) | -24.93 – 16.73 | 0.70, 0.02 |
| Gaze duration |  |  | -1.24 (1.64) | -4.52 – 2.04 | 0.45, 0.14 |
| SSRT |  |  | -0.17 (0.16) | -0.49 – 0.16 | 0.31, 0.05 |
| Stream liking |  |  | 0.18 (0.25) | -0.31 – 0.67 | 0.46, 0.20 |
| VGLSP total |  |  | 2.36 (0.80)* | 0.77 – 3.95 | **0.004, 0.38** |
| **Step two** | .02 | (4, 61) 0.33 |  |  |  |
| Condition*gaze duration |  |  | 0.16 (2.72) | -5.28 – 5.60 | 0.95, 0.04 |
| Condition*SSRT |  |  | 0.18 (0.21) | -0.24 – 0.59 | 0.39, 0.09 |
| Gaze duration*SSRT |  |  | 0.03 (0.03) | -0.04 – 0.10 | 0.41, 0.06 |
| Condition*gaze duration*SSRT |  |  | -0.04 (0.05) | -0.15 – 0.07 | 0.45, 0.10 |

*p <.05. The overall model was non-significant (R^2^ = 0.18, F(9, 61) = 1.52, p = 0.16).

**Table A2**: Model 2, predictors of overall intake (with participants aged 19 and over excluded)

|  | Cumulative | | Simultaneous | |  |
| --- | --- | --- | --- | --- | --- |
|  | R^2^ change | F- change | B (SE) | CIs | *p*, Cohen’s *f* |
| Step one | .08 | (5, 64) 1.17 |  |  |  |
| Condition |  |  | 4.58 (19.14) | -33.71 – 42.87 | 0.81, 0.08 |
| Gaze duration |  |  | 0.63 (2.98) | -5.33 – 6.58 | 0.83, 0.02 |
| SSRT |  |  | -0.40 (0.31) | -1.01 – 0.22 | 0.20, 0.12 |
| Gender |  |  | -51.56 (22.81)* | -97.19 - -5.93 | **0.03, 0.24** |
| Fortnite familiarity |  |  | -0.05 (0.41) | -0.86 – 0.76 | 0.90, 0.13 |
| Step two | .05 | (4, 60) 0.93 |  |  |  |
| Condition*gaze duration |  |  | -0.09 (5.08) | -10.25 – 10.08 | 0.99, 0.02 |
| Condition*SSRT |  |  | 0.28 (0.37) | -0.46 – 1.03 | 0.45, 0.10 |
| Gaze duration*SSRT |  |  | 0.03 (0.06) | -0.10 – 0.15 | 0.67, 0.09 |
| Condition*gaze duration*SSRT |  |  | -0.15 (0.09) | -0.34 – 0.04 | 0.11, 0.21 |

*p <.05. The overall model was non-significant (R^2^ = 0.14, F(9, 60) = 1.06, p = 0.41).

**Table A3**: Model 1, predictors of Doritos intake (with aim guessers excluded)

|  | Cumulative | | Simultaneous | |  |
| --- | --- | --- | --- | --- | --- |
|  | R^2^ change | F- change | B (SE) | CIs | *p*, Cohen’s *f* |
| Step one | .17 | **(5, 66) 2.68*** |  |  |  |
| Condition |  |  | -1.67 (10.83) | -23.32 – 19.97 | 0.88, 0.06 |
| Gaze duration |  |  | -0.41 (1.57) | -3.55 – 2.72 | 0.79, 0.08 |
| SSRT |  |  | -0.09 (0.15) | -0.40 – 0.21 | 0.54, 0.03 |
| Stream liking |  |  | 0.33 (0.24) | -0.14 – 0.80 | 0.17, 0.27 |
| VGLSP total |  |  | 2.13 (0.84)* | 0.45 – 3.81 | **0.01, 0.35** |
| Step two | .01 | (4, 62) 0.17 |  |  |  |
| Condition*gaze duration |  |  | -0.37 (2.70) | -5.76 – 5.03 | 0.89, 0.00 |
| Condition*SSRT |  |  | 0.09 (0.20) | -0.31 – 0.49 | 0.65, 0.04 |
| Gaze duration*SSRT |  |  | 0.02 (0.03) | -0.04 – 0.088 | 0.48, 0.05 |
| Condition*gaze duration*SSRT |  |  | -0.04 (0.05) | -0.14 – 0.07 | 0.50, 0.09 |

*p <.05. The overall model was non-significant (R^2^ = 0.18, F(9, 62) = 1.49, p = 0.17).

**Table A4**: Model 2, predictors of overall intake (with aim guessers excluded)

|  | Cumulative | | Simultaneous | |  |
| --- | --- | --- | --- | --- | --- |
|  | R^2^ change | F- change | B (SE) | CIs | *p*, Cohen’s *f* |
| Step one | .09 | (5, 66) 1.33 |  |  |  |
| Condition |  |  | 5.33 (19.54) | -33.74 – 44.40 | 0.79, 0.07 |
| Gaze duration |  |  | 2.47 (2.77) | -3.07 – 8.01 | 0.38, 0.09 |
| SSRT |  |  | -0.24 (0.28) | -0.81 – 0.33 | 0.40, 0.08 |
| Gender |  |  | -45.47(22.38)* | -90.20 - -0.74 | **0.05, 0.21** |
| Fortnite familiarity |  |  | 0.25 (0.41) | -0.57 – 1.06 | 0.55, 0.20 |
| Step two | .04 | (4, 62) 0.74 |  |  |  |
| Condition*gaze duration |  |  | -2.18 (4.94) | -12.06 – 7.69 | 0.66, 0.04 |
| Condition*SSRT |  |  | 0.16 (0.03) | -0.56 – 0.88 | 0.66, 0.05 |
| Gaze duration*SSRT |  |  | 0.03 (0.06) | -0.09 – 0.14 | 0.670.08 |
| Condition*gaze duration*SSRT |  |  | -0.14 (0.09) | -0.33 – 0.04 | 0.13, 0.19 |

*p <.05. The overall model was non-significant (R^2^ = 0.13, F(9, 62) = 1.06, p = 0.41).

**Figure A7**: Participant flow


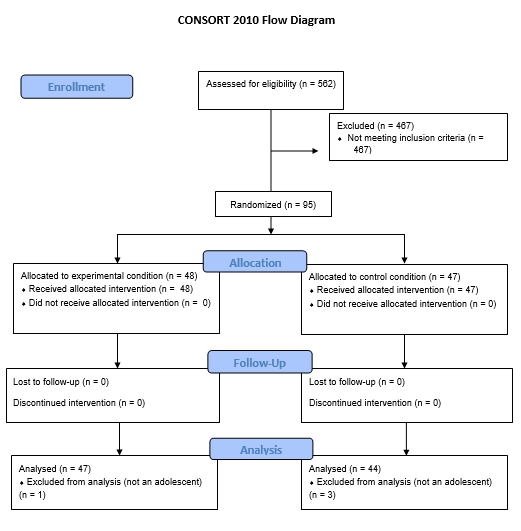

Supplement: Evans et al. supplementary material [file S1368980025100487sup001.docx]
